# Human umbilical cord mesenchymal stem cell-derived exosomes promote microcirculation in aged diabetic mice by TGF-β1 signaling pathway

**DOI:** 10.1186/s13098-023-01191-x

**Published:** 2023-11-15

**Authors:** Weijian Fan, Mengdie Zhou, Shaoqiu Zheng, Yang Liu, Songsong Pan, Peng Guo, Minjie Xu, Chao Hu, Anle Ding, Zan Wang, Shiwu Yin, Keqiang Zuo, Xiaoyun Xie

**Affiliations:** 1 Department of Interventional & Vascular Surgery, Hefei Second People’s Hospital, Hefei Hospital Affiliated to Anhui Medical University, Anhui, 230011 China; 2 Shanghai Tenth People’s Hospital, Tongji University School of Medicine, Shanghai, 200072 China; 3 Department of Urinary Surgery Shanghai Pudong New District Zhoupu Hospital, Shanghai, 200100 China; 4 Department of Geriatrics, Tongji Hospital, Tongji University School of Medicine, Shanghai, 200065 China; 5 Department of Vascular Surgery, The Fifth Affiliated Hospital of ZhengZhou University, ZhengZhou, 450052 China; 6 AnHui University of Science and Technology, Huainan, 232001 China; 7 Geriatric department, Shanghai YangZhi Rehabilitation Hospital (Shanghai Sunshine Rehabilitation Center), School of Medicine, Tongji University, Shanghai, 201600 China

**Keywords:** Diabetic peripheral angiopathy, Exosomes, CD105, TGF-β1, Microvascular

## Abstract

**Background:**

Microvascular dysfunction is one of the most common pathological characteristics in Type 2 diabetes. Human mesenchymal stem cell-derived exosomes (hUCMSCs-Exo) have diverse functions in improving microcirculation; however, the molecular mechanism of hUCMSCs-Exo in regulating burn-induced inflammation is not well understood.

**Methods:**

hUCMSCs-Exo were extracted by hypervelocity centrifugation method, and exosome morphology was observed by transmission electron microscopy, exosome diameter distribution was detected by particle size analysis, and exosome specific proteins were identified by Western blot.2. DB/DB mice were randomly divided into exosomes group and PBS group. Exosomes and PBS were injected into the tail vein, respectively, and the calf muscle tissue was taken 28 days later. 0.5% Evans blue fluorescence assessment microvascular permeability. The expression of CD31 was detected by immunofluorescence.The morphology and function of microvessels in muscle tissue of lower limbs was evaluated by transmission electron microscopy.3. TMT proteomics was used to detect the changes of differential protein expression in lower limb muscle tissues of the PBS group and the exosome group, and data analysis was performed to screen key signal molecules and their involved biological pathways. Key signal molecules CD105 were verified by Western blot. The expression of TGF-β1 in exosomes were evaluated by Western blot.

**Results:**

Electron microscopy showed that hUCMSCs-Exo presented a uniform vesicle structure, and NTA showed that its diameter was about 160 nm. Western blot showed positive expression of specific proteins CD9, CD81 and TSG101 on exosomes.2. There is no significant change in blood glucose and body weight before and after the exosome treatment. The exosome group can significantly reduce the exudation of Evans blue. Compared with the PBS group. Meanwhile, CD31 immunofluorescence showed that the red fluorescence of exosome treatment was significantly increased, which was higher than that of PBS group. Transmission electron microscopy showed smooth capillary lumen and smooth and complete surface of endothelial cells in the exosome group, while narrow capillary lumen and fingerlike protrusion of endothelial cells in the PBS group.3.Quantitative analysis of TMT proteomics showed that there were 82 differential proteins, including 49 down-regulated proteins and 33 up-regulated proteins. Go enrichment analysis showed that the differential proteins were involved in molecular function, biological process, cell components,among which CD105 was one of the up-regulated proteins. Through literature search, CD105 was found to be related to endothelial cell proliferation. Therefore, this study verified the changes of CD105 in the exosome group, and it was used as the mechanism study of this study. 4. Western blot analysis showed that the expression of CD105 protein in lower limb muscle tissue of exosome group was significantly increased compared with that of PBS group. Based on the fact that CD105 is a component of the TGF-β1 receptor complex and exosomes are rich in growth factors and cytokines, this study further examined the expression of TGF-β1 in exosomes, and the results showed that exosomes had high expression of TGF-β1.

**Conclusion:**

By improving the integrity of microvascular endothelial cells, hUCMSCs-Exo can improve the permeability of microvessels in diabetic lower muscle tissue, further promote the proliferation of lower limb muscle cells and inhibit the apoptosis of tissue cells. The mechanism may be associated with exosomes rich in TGF-β1, which is likely to promote endothelial cell proliferation and improve permeability through binding to the endothelial CD105/TβR-II receptor complex, while promoting angiogenesis and protecting skeletal muscle cells from apoptosis.

**Supplementary Information:**

The online version contains supplementary material available at 10.1186/s13098-023-01191-x.

## Introduction

With the improvement of life quality and lifestyle, the morbidity and mortality of Diabetes mellitus (DM) have also increased. About 475 million people suffered from diabetes in 2017, and DM had become the 10th leading cause of death in the world [1]. DM is characterized by hyperglycemia. And frequent and long-term hyperglycemia can lead to a host of microvascular complications [2]. Currently, it remains a great challenge to prevent and protect against diabetes-induced microvascular injury. The reported studies had found that human umbilical cord mesenchymal stem cells (hUCMSCs) could take the edge off diabetic microangiopathy [3]. hUCMSCs are primitive cells with the potential of self-replication and multidirectional differentiation, which are of great importance for angiogenesis and regulation of vascular dysfunction [4, 5].

Recently, many studies have found that the main function of MSCs comes from their secretion function, but not their differentiation ability [6]. Exosomes are nanosized extracellular vesicles (EVs) that show great prospect in reducing inflammation and immunoregulation as MSCs [7]. Exosomes can affect protein expression in target cells through functional transfer between cells. Exosomes are 10 to 100 nm in size,which comprise mRNA, miRNA molecules, lipids,and proteins. Exosomes can influence the expression of protein in target cells through functional transfer among cells. Some studies have shown that exosomes can promote endothelial cell proliferation and microvascular repair, making them a potential effective alternative to MSCs transplantation therapy [8]. However, the mechanisms involved in hUCMSCs in excessive inflammation of diabetic microangiopathy are not yet explained. Here we hypothesized that exosomes may improve the microcirculation by regulating target proteins in certain signaling pathways.

The aim of this study was to investigate the therapeutic effect of hUCMSCs-Exo on microangiopathy in diabetic mice and explore its potential mechanism. Exosomes do not contain nuclear structures, can neither self-replicate nor mutate, and can only mediate cell-to-cell signaling, thus avoiding some adverse events caused by direct transplantation of stem cells. This paper provides a theoretical basis for the clinical application of hUCMSCs-Exo in human diabetic microangiopathy.

## Materials and methods

### Exosome isolation

Under aseptic conditions, 10 cm of umbilical cords of healthy newborns were taken from the operating room and placed in a sterile, wide-mouth bottle containing a mixture of 300 ml PBS and double antibody. After the pretreatment in the ultra-clean studio, the umbilical cord was removed and placed in a sterile petri dish containing PBS on the ultra-clean workbench. Wash until PBS is clear, cut the Walton glue separated from umbilical cord to 1 cc fragments with tissue shears, put 1 cc into T-25 cm^2^ inoculation flask, add 6 ml of medium containing FBS, and culture in a 5%C0_2_ cell incubator at 37 ℃. When the fusion of cells reached 70–80%, the hUCMSCs of the P4 generation with good growth status were selected and cultured in the high-glucose DMEM/F12 medium containing 10% FBS. When the fusion of cells reached 60–80%, the serum-free medium was changed for culture. After 72 h, the sample medium was collected. The obtained supernatant was then passed through a 0.22 μm disposable filter and collected. When the hUCMSCs had reached 60–80% confluence, exosomes were isolated from the collected medium by hypercentrifugation. Supernatant collected from cultured mesenchymal stem cells was centrifuged at 2000 g for 20 min to remove large fragments and dead cells (Optima L-100 XP Ultracentrifuge, Backman, USA), The upper liquid was centrifuged at 10,000 g for 30 min, and the filtered liquid was centrifuged at 100000 g for 80 min. Finally, the precipitated exosomes were collected. The exosomes were stored at − 80 °C. To quantify the isolated exosomes, The protein content of exosomes was examined by BCA assay. Western blotting was used to examine specific exosome surface markers, including CD9 (ab92726; Abcam), CD63(ab13404;Abcam) and TSG101(ab125011; Abcam).

### Transmission electron microscopy (TEM) analysis of hUCMSCs-Exo

The exosomes samples were dropped on the carbon supporting membrane mesh for 3–5 min, and then the excess of liquid was removed with filter paper. Drop 2% phosphotungstic acid (Servicebio) on the copper grid of the carbon supporting membrane for 2–3 min, then used a piece of filter paper to wipe off excess solution. Exosome morphology was observed and the images were acquired and analyzed under TEM (HT7700, hitachi).

### Particle size analysis of exosomes

Take 5 μl of exosomes separated by hypercentrifugation and dilute them to 1 mL. Pipette the samples gently for thorough mixture, then transfer them to designated sample tank, place the tank into NS300 (Malvern company) nanometer particle size analyzer to test the particle size range of the exosomes. The parameters as follows: particle size range of 50–200 nm, molecular weight range of 1000–20107, Dalton temperature range of 25 ℃, 4.0 mW He–Ne laser, wavelength at 633 nm. Each sample was analyzed continuously at least three times (3–5 times). Data was analyzed using Brookhaven Instruments’ Nanosight NTA data analysis software.

### Western blotting analysis

The protein concentration of the exosomes sample was measured by BCA method (Beyotime Biotechnology), 20 μg protein was used for SDS-PAGE (Beyotime Biotechnology). 10 μl RIPA lysate (Beyotime Biotechnology) was added to the same volume of PBS resuspended UCMSCs exosome. The supernatant was extracted after centrifugation at 10000 g at 4 ℃ for 5 min. The supernatant was mixed with Loading Buffer at a volume ratio of 5:1, and the mixture was boiled at 100 ℃ for 15 min in a constant temperature metal bath. Protein samples and protein Maker (Prestained).

Color Protein Ladder15–120 kD, Beyotime Biotechnology) were added into the 12% SDS-PAGE gel hole in sequence. Run the glue at 80 V until the sample runs out of the concentrated glue, then transfer the glue at 100 V to the bromophenol blue to the rubber bottom (EPS-300, Tianneng Shanghai). The membrane was transferred at 200 mA, and the time of membrane transfer was determined according to the size of the target protein. The general rule was 1 min for 1kd. After the transfer, the PVDF membrane was taken out and soaked in 5% skimmed milk TBST, face up, for 1.5 h. Dilute the primary antibody with TBST diluent buffer containing 5% BSA according to the instructions, Then the film was incubated overnight at 4 °C and washed with TBST for 15 min (3 times in total). Incubate at room temperature in secondary antibody dilution (using 5% skimmed milk powder TBST) for 1 h, then wash the membrane with TBST for 10 min (3 times). ECL reagent (A: B = 40:1) was then used to incubate the membrane, and imaging was detected by Typhoon scan (Typhoon parameters: wavelength 473 nm, voltage 485 V).

### Grouping and treatment of experimental mice

8 week-old male DB/DB mice (weighing 210 ± 20 g, No. SCXK2014-0004) were purchased from south model company, and were fed at 26 ℃, 38.5% humidity, 12 h of light, and 12 h of darkness cycle (7:30 a.m. to 7:30 p.m.) in the SPF environment. The Animal Ethics Committee of Tongji University approved the protocol for mice. 13 mice were randomly tested for blood glucose and given insulin weekly. Then the DB/DB mice were randomly divided into two groups at 26 weeks: hUCMSC-exosome group (n = 8), injection with 100 μg (Protein concentration) hUCMSC-exosomes suspended in 0.1 ml PBS; PBS group (n = 5), injection with 0.1 ml PBS. 28 days after injection, all mice were sacrificed by cervical dislocation, and the calf tibialis anterior muscle group and gastrocnemius muscle group were completely isolated, which were divided into 3 parts and put into electron microscopy solution, 4% formaldehyde fixation solution and liquid nitrogen for subsequent experiments. These experiments include immunohistochemistry, transmission electron microscopy to assess microvascular morphological changes, 0.5% Evans blue immunofluorescence to assess microvascular permeability, and proteomics to detect differential protein expression changes (Fig. 7).

### Immunofluorescence staining

The paraffin tissue sections were successively soaked in xylene, anhydrous ethanol, alcohol of different concentrations and distilled water. Then, the sections were soaked in EDTA antigenic repair solution (PH9.0), boiled and cooled naturally. After washing and sealing, the prepared primary antibody CD31 (1: 200 ab222783 Abcam) was dropped onto the sections. After incubating the secondary antibodies and washing, the tablets were sealed with anti-fluorescence quenching sealing tablets. The images were observed and collected under a Nikon inverted fluorescence microscope.

### Microvascular permeability analysis

After injected with 0.5% EB (1.5 ml/kg) into the caudal vein for 1 h, the mice were anesthetized. The calf muscle tissue was collected and fixed in 4% paraformaldehyde solution for 12 h. After automatic dehydrating and embedding, paraffin pathological sections (anti-slide slides) were made. The slices were roasted for 2.5 h, dewaxed with gradient alcohol, hydrated, and sealed with direct glycerin. The distribution of EB in muscle tissue was immediately observed under excitation light of 540 nm using fluorescence microscope. The average fluorescence intensity was evaluated by Image J under the same exposure conditions.

### Transmission electron microscopy

The muscle tissues in the electron microscope fixative solution were fixed in turn, rinsed and dehydrated. Pure 812 embedding agent was prepared, and the sample was inserted into the embedding plate. After oven at 37 ℃ for one night, the temperature was raised to 60 ℃ for 48 h. The samples were cut into 60–80 nm sections using an ultra-thin slicer, and the sections were stained and observed under a transmission electron microscope.

### Proteomic analysis

#### Protein extraction and labeling

SDT lysis method was used to extract protein from muscle tissue, and BCA (Bio-Rad, USA) method was used for protein quantification. Filter Aided Proteome Preparation (FASP) method was used for trypsin enzymolysis, and the peptides were desalted using the C18 Cartridge. After lyophilized, the peptides were added to 40μL 0.1% formic acid solution for resolution. 100 μg of peptides were taken from each sample and labeled by Thermo’s TMT labeling kit. (Thermo Scientific).

#### LC–MS/MS analysis

Easy NLC with nanositre flow rate and HPLC liquid phase system was used to separate the samples. After chromatographic separation, the samples were analyzed by Q-Exactive mass spectrometry. For identified and quantitative analysis, original data of mass spectrometry were transferred to Mascot2.2 and Proteome Discoverer1.4.

#### Bioinformatic analysis

For hierarchical clustering analysis, we used Cluster 3.0 and Java Treeview software. Briefly, the quantitative information of the target protein set is normalized to the interval of (− 1,1). As for the hierarchical clustering heatmap, we used the ComplexHeatMap R package (R Version 3.4) to simultaneously classify the expression levels of the samples and proteins (distance algorithm: Euclide, linkage: Average linkage). The sub-cellular location prediction was carried out using Cello (http://cello.life.nctu.edu.tw/), which was modeled by multi-class SVM classification system. We used Fisher's Exact Test to compare the distribution of the target protein set and the total protein set in GO classification or KEGG pathway. Blast2GO was used to annotate the target protein set, which can be summarized in four steps: Blast, Mapping, Annotation, and Annotation Augmentation at InterProScan. As for KEGG pathway, KAAS (KEGG Automatic Annotation Server) software was used to annotate the target protein set.

### Statistical analysis

Image J was used to quantitatively evaluate the mean fluorescence intensity of Evans blue staining and CD31 immunofluorescence, and the analyzed data were presented in a bar chart. Images were taken from the calf muscles for each analysis group and were obtained using the same exposure Settings. The data were analyzed by SPSS 20.0 statistical software. Significant differences between the PBS and exosome groups were evaluated by independent T-test. All data are expressed in X̅ ± SD, *P* < 0.05 is considered to have significant difference.

## Result

### Changes in blood glucose and body weight of mice before and after treatment

**Fig. 1:**
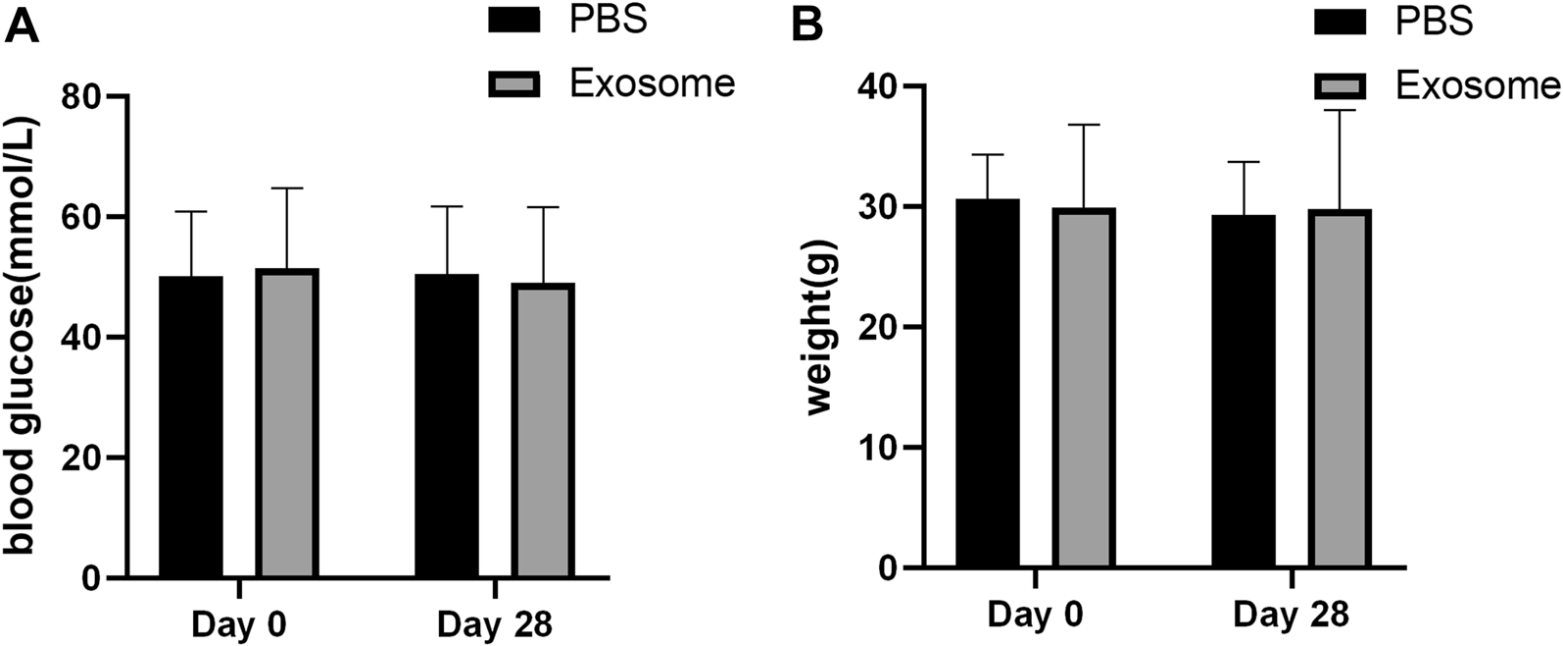
Changes in blood glucose and weight before and after treatment in EXO PBS groups **A** and **B** values are respectively in the two groups before and after treatment for 28 days random blood glucose, weight change, Expressed in ± SD

There were no significant differences in blood glucose or body weight between the exosome group and PBS group. As shown in Fig. 1, Blood glucose and body weight in mice were recorded at the time of treatment and 28 days after treatment, but there was no statistically significant difference, which may indicate that exosomes have no significant effect on blood glucose and body weight, or due to the differences in the injection dose or experimental duration.

### Identification of exosomes of hUCMSCs

**Fig. 2:**
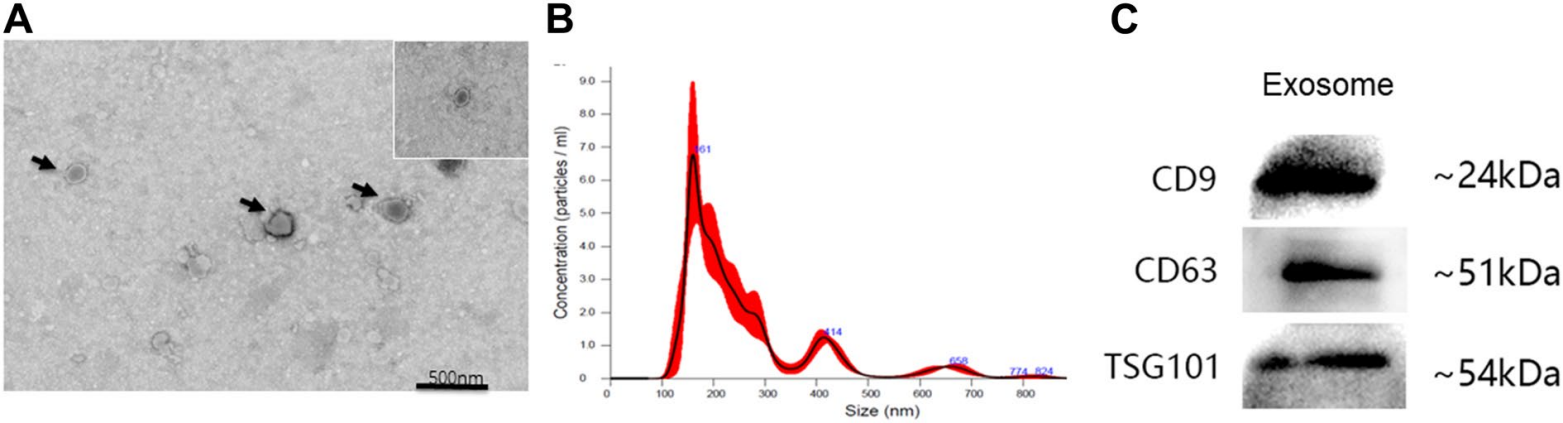
Identification of exosomes of hUCMSCs **A** Morhology of exosomes observed by TEM. **B** Aanalysis of nanoparticle size distribution by NS300 (Malvin) nanoparticle analyzer. **C** High expression of CD9 and TSG101 BY Western blot

Transmission electron microscopy and nanometer particle size analysis revealed that exosomes were spherical vesicles and typical discoid vesicles, and its particle size distribution ranges from 30 to 200 nm. Specific exosome surface markers including CD9, CD63, and TSG101 were positive based on Western blotting results, further confirming the presence of exosomes (Fig. 2).

### Tissue fluorescence imaging with evans blue staining

**Fig. 3:**
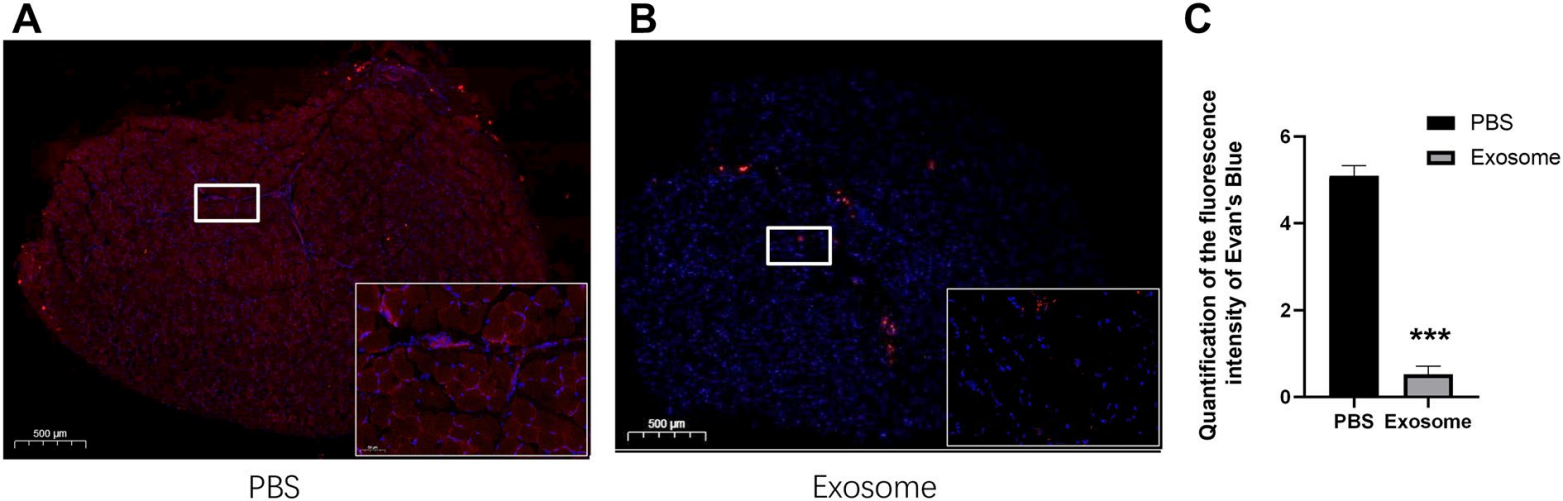
Exosome therapy reduced the microvascular permeability of lower limb muscles in diabetic mice. **A** Typical fluorescence images of Evans blue dye in the PBS group after 28 days of treatment. **B** Typical flurescent images of Evans Blue dye in the exosme group after 28 days treatment. **C** Quantitative analysis of fluorescence intensity after 28 days of treatment. *p < 0.05 compared with PBS group

Evans blue dyes can be quantitatively combined with plasma albumin, and the changes of vascular walls permeability can be measured by colorimetric method. hUCMSCs-Exo restored microcirculation permeability in the lower extremity. Figure 3(B) showed that the exosome treatment significantly reduced Evans blue exudation on day 28. Quantitative analysis of fluorescence intensity (Fig. 3C), The fluorescence intensity of EB dye exudation in Exosome group was significantly decreased. These indicate that hUCMSCs-Exo could effectively improve the permeability of microcirculation.

### Immunofluorescence staining of CD31 in muscle vessels and electron microscopy of capillary ultrastructure

**Fig. 4:**
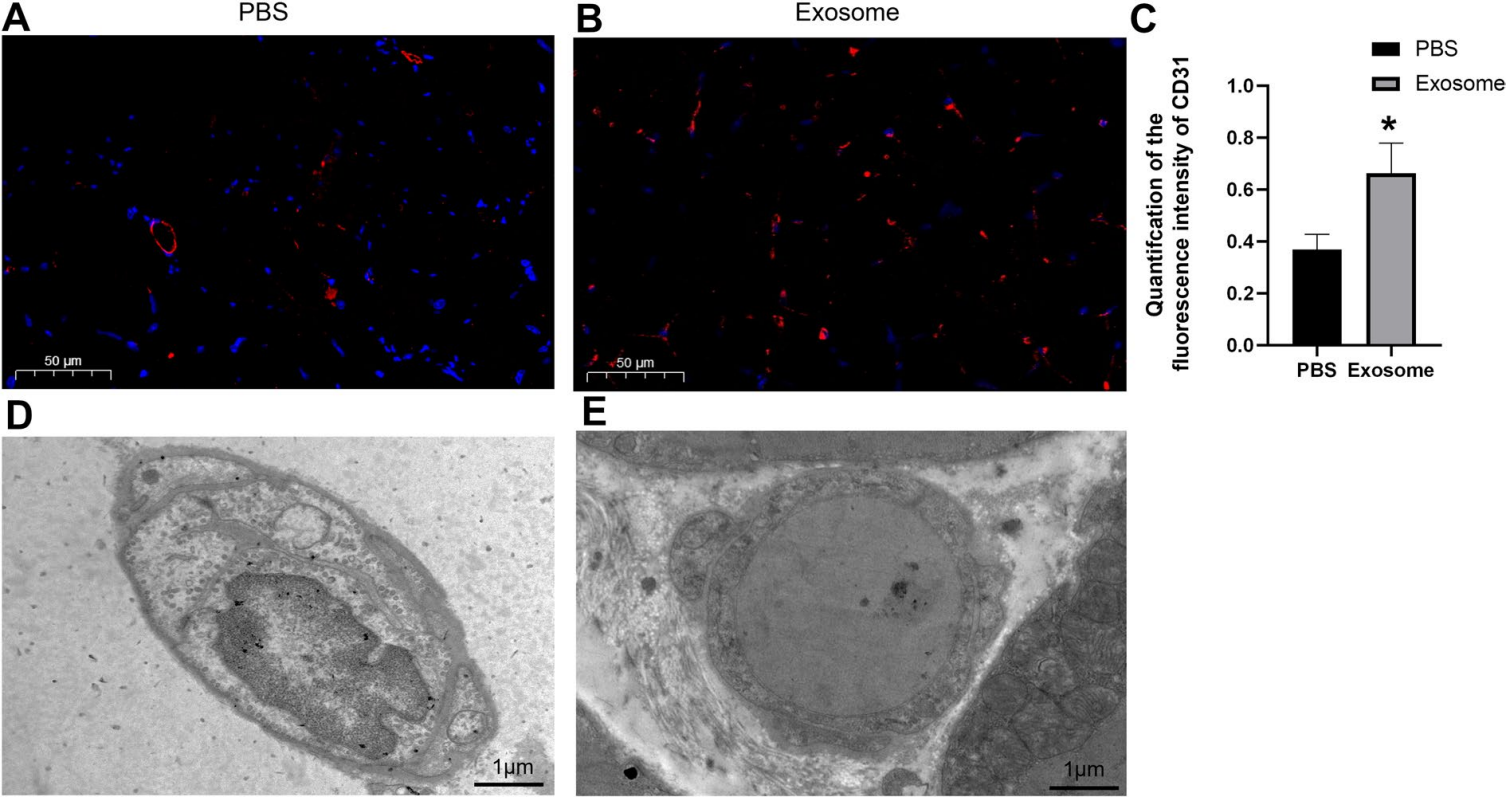
Immunofluorescence staining of CD31 in muscle vessels and electron microscopy of capillaryultrastructure. The CD31 immunofluorescence (red) region was increased in the exosome treatment group **B** compared to the PBS group **A**. **C** Quantitative analysis of CD31 fluorescence intensity on the 28 th day vs PBS, *P < 0.05. **D**. In PBS group the capillary lumen was narrow, the gap of endothelial cell was widened, and fingerlike protrusion and the pinocytotic vesicles in endothelial cells were increased and the mitochondria swelling, cristae shedding, vacuolar changes, intearity destruction. Endothelial space widened. **E**. In the exosome group.capillary lumen was smooth the gap of endothelial cells was nomal. The surface of endothelial cells was smooth and complete

Immunofluorescence results showed that the amount and density of red fluorescence in the PBS group were sparse (Fig. 4A), The results are calculated by ImageJ, while the density of red fluorescence in the exosome group (Fig. 4C) were increased (*P* < 0.05). The results of electron microscopy demonstrated that in the PBS group, the capillary lumen was narrow, the gap of endothelial cell was widened, finger-like protrusion and the pinocytotic vesicles in endothelial cells were increased, and mitochondria were swollen, cristae were shed, vacuoles were changed, and the intercellular space of endothelial cells was widened (Fig. 4D). While in the exosome group, capillary lumen was smooth, the gap of endothelial cells was normal, the surface of endothelial cells was smooth and complete (Fig. 4E).

### Heat map analysis of the differential proteins

**Fig. 5:**
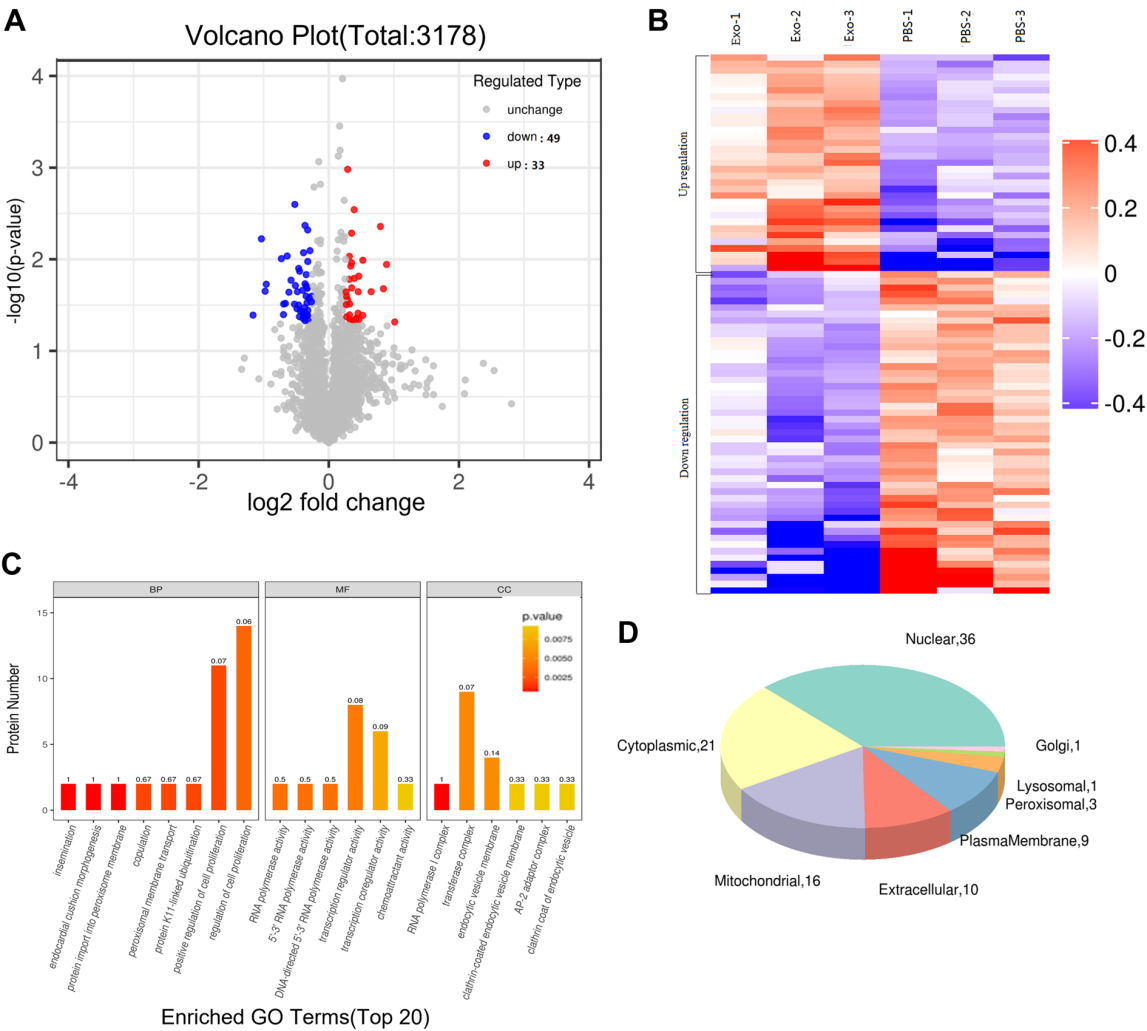
Quantitative protemics by TMT and biochemistry analysis. **A** ITRAQ based protemic maps of 3178 differentially expressed proteins, including 49 down-regulated proteins (with a 0.83-fold truncation) and 33 up-regulated proteins (with a1.2-fold truncation),(p<0.05). **B**. Cluster heat map analysis of differential proteins (49 down-regulated and 33 up-regulated proteins) corresponding to the volcano map. **C**. All differential proteins and the op 20 enriched GO annotated GO classifications (biological processes molecular functions and celcomponents). **D** Pie chart of subcellular localization of differential proteins

To further investigate the effect of exosomes on muscle tissues of DB/DB mice, the hind limb muscle tissue of mice was quantitatively analyzed by tandem mass tag (TMT) technology. This technique uses multiple isotope tags to covalently bind with the amino group of the peptide segment, which enables simultaneous qualitative and quantitative analysis of proteins in different samples, and is widely used in the analysis of differentially expressed proteins. Results from the TMT technology-based proteomic volcano map (Fig. 5A) showed 82 differential proteins, of which there are 49 down-regulated proteins (cut-off expression variation 0.83) and 33 up-regulated proteins (cut-off expression variation 1.2). These differential proteins were indicated in the cluster heat map analysis, showing good intra-group and inter-group parallelism (Additional file 1: Fig. 5B).

### GO classification of differential proteins and enriched GO terms

According to the annotation by GO analysis, the cellular components, molecular functions and biological process of 82 differentially expressed proteins in the muscle tissues of mice between the PBS group and the exosome group were classified. The analysis results showed that the enrichment of molecular functions mainly involved the activities of transcriptional coactivators and transcriptional coregulators. The enrichment of cell components mainly involved transferase complex, endocytic vesicle membrane and RNA polymerase I complex. Enrichment of biological processes mainly involves regulation of cell proliferation and positive regulation of cell proliferation (Fig. 5C).

### Subcellular localization analysis

The specific positions of 82 differential proteins in cells were analyzed, and the analysis results showed that 36 of the 82 differential proteins were located in the nucleus with 21 in the cytoplasm, 16 in mitochondria and 10 in extracellular (Fig. 5D).

### Identification of CD105 and TGF-β1 proteins

**Fig. 6:**
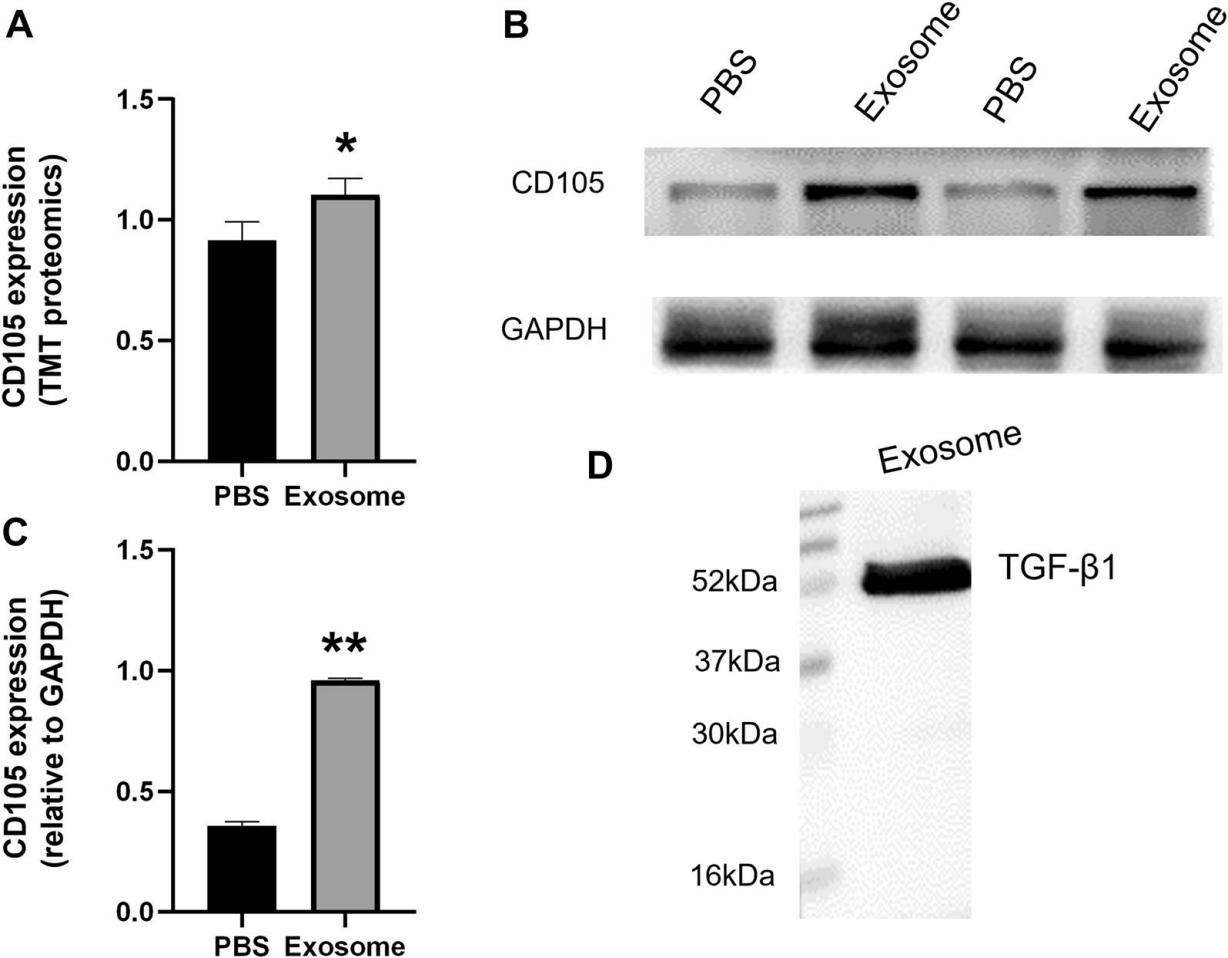
Identification of CD105 and TGF-β1 proteins. **A** Differential analysis o CD105 detected by TMT proteomics. **B** CD105 expression in exosome group in the the exosome group was increased by western blot. *n* = 4, vs PBS. **C** CD105 expression of muscle tissue (*p < 0.05 vs PBS). **D**. The expression of TGF-β1 protein exosomes by Western blot

The expression of CD105 in muscle tissue and the expression of TGF-β1 in exosomes were determined by Western blot. The results showed that the expression of CD105 in the exosome group was significantly higher than that in the PBS group by TMT proteomics and Western blot (Fig. 6A, *P* < 0.05). The exosomes contained the cytokine TGF-β1 with a molecular weight of about 52 kDa (Fig. 6D).

## Discussion

This present study confirmed that hUCMSCs-Exo can significantly promote endothelial cell proliferation, improve microvascular permeability, promote neovascularization, and protect skeletal muscle cells from apoptosis in DB/DB mice. Meanwhile, the results of TMT technology indicated that exosomes might improve microvascular disorders caused by diabetes through TGF-β1 signaling pathways.

The most common complication of type 2 diabetes mellitus is endothelial cell dysfunction caused by chronic hyperglycemia [9]. The main pathophysiological basis is that endothelial cell structure and function damage leads to increased vascular permeability. Previous studies have demonstrated that hUCMSCs may enhance the Tube-Formation ability of endothelial cells and promote angiogenesi [10]. Paracrine is considered to be the main underlying mechanism behind stem cell therapy [11]. Recent research has shown that hUCMSCs-Exo can effectively enhance angiogenesis and promote skin wound healing [12], promote cartilage repair [13] and bone regeneration [14], protect cardiomyocytes from acute myocardial infarction [15], and inhibit the infiltration of inflammatory cells, improve microcirculation and prevent kidney injury [16]. In the present study, we found that the fluorescence intensity of EB dye exuding in the immunofluorescence region in the PBS group was significantly higher than that in the exosome treatment group, which is consistent with the work of Yuan et al [17]. Electron microscopic images of microvascular suggesting that exosomes can promote angiogenesis, repair endothelial cell damage, and improve microvascular permeability. The therapeutic effect of hUMSCs-Exo was due in part to the direct stimulation of endothelial cells.

A number of substances delivered by exosomes have been found to play an active role in angiogenesis [18, 19]. Recent studies have confirmed that exosome-mediated miRNA can promote the proliferation and metastasis of tumor cells through the overexpression of TGF-β1 [20, 21]. In our study, according to Gene Ontology (GO) classification, the analysis results showed that the enrichment of molecular functions mainly involved the activities of transcriptional coactivators and transcriptional coregulators. The enrichment of cell components mainly involved transferase complex, endocytic vesicle membrane, RNA polymerase I complex, etc. Enrichment of biological processes mainly involves regulation of cell proliferation, positive regulation of cell proliferation, etc. In GO enrichment analysis, a higher quantity of identified proteins concentrated in cellular component (especially golgi, cytoplasmic domain, mitochondria), indicating that the intracellular region has undergone obvious and essential changes. Differential protein spots on this signaling pathway can serve as key targets for exosomes to function. We screened out a specific protein CD105 (Endoglin) related to endothelial cell proliferation and repair, which is a marker of endothelial cell proliferation and neovascularization [22]. CD105 is an accessory transmembrane glycoprotein of the transforming growth factor-β (TGF-β) receptor system,and expressed on activated vascular endothelial cells [23]. CD105 plays a great important role in endothelial cell proliferation and neovascularization [24]. In general, TGF-β1 activates Smad1/5/8 signaling pathway through CD105 and promotes endothelial cell migration, proliferation, and tubular formation [25]. In conclusion, these data suggest that the improved capillary permeability was due to the role of promoting angiogenesis and angiostatic factors in hUCMSCs-Exo.

We only observed the short-term therapeutic effect of exosomes on microcirculation in diabetic mice. Other functions of exosomes, such as anti-fibrosis [26], anti-inflammatory [27] and immunoregulatory function [28], are still to be studied. In addition, TGF-β1 pathway still needs to be further validated. Whether these exosomes play their role by regulating other signaling pathways in the receptor cells remains to be further investigated. The proteomic identification of CD105 as a regulator of endothelial cell proliferation can further understand the potential mechanisms of neovascularization and tissue repair. The shortcoming of this study is that no experimental study was conducted to compare the cellular level and CD105 antagonist group, which will be the starting point for further research.

**Fig. 7:**
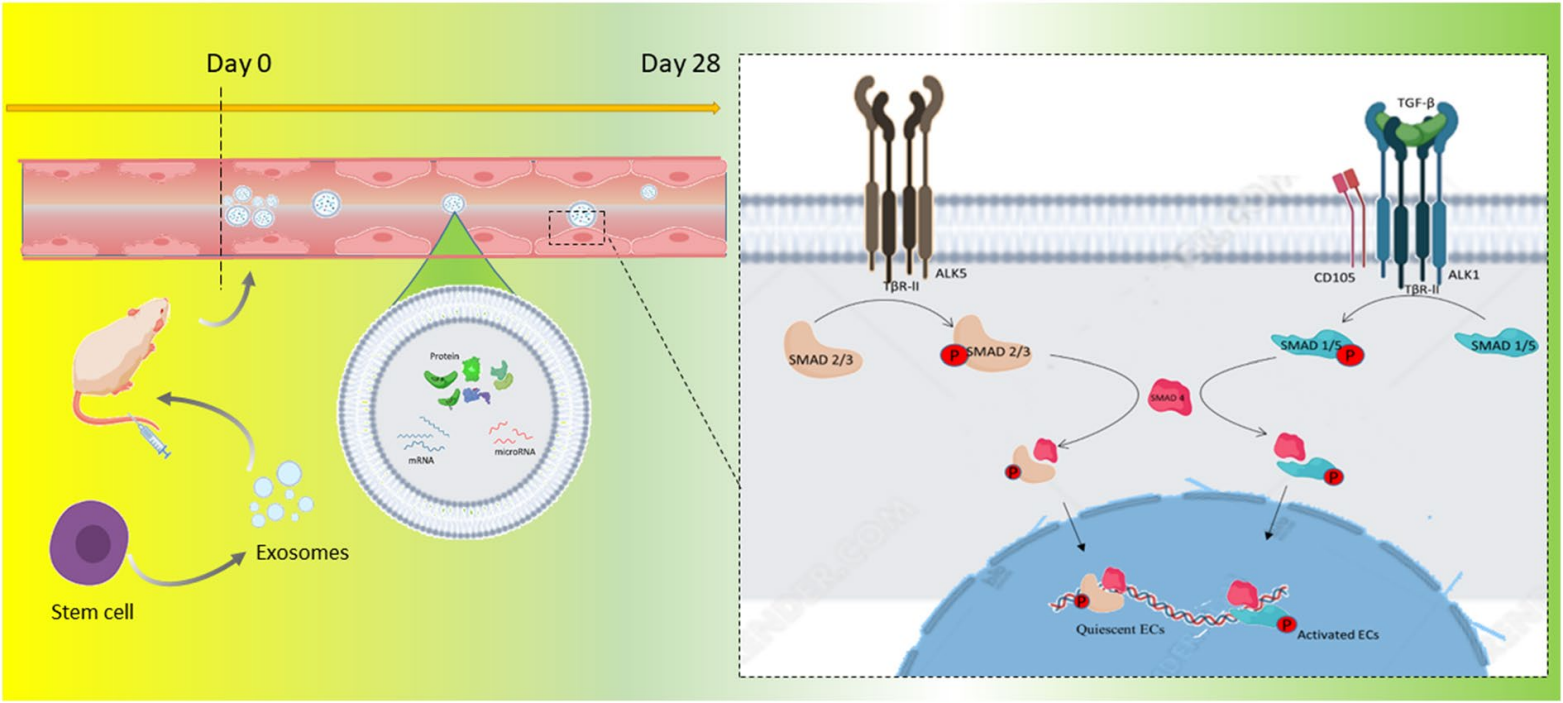
Therapeutic mechanism of hUCMSCs after increased microcirculation permability in diabetic mice. Exosomes obtained by overspeed centrifugation were injected through the caudel vein, binding to CD105/TβR-II receptor complexes on the surface of endothelial cell membrane in mice through TGF-β cytokines in exosomes, activating downstream signals, endothelial cell activation and proliferation, endothelial space narrowing, permability reduction, and angiogenesis

We hypothesize that hUCMSCs-Exo may activate the downstream signaling of endothelial cell proliferation through TGF-β1 and endothelial cell membrane surface CD105/TβR-II receptor complexes, thus strengthening adherent junction and inhibiting the formation of endothelial gaps, thereby reducing micro-vessel permeability and angiogenesis. hUCMSCs-Exo are not only a new therapeutic strategy for angiogenesis, but also bring challenges and opportunities.The experimental procedure and treatment mechanism are shown in the Fig. 7.

### Supplementary Information

**Additional file 1:** **Fig. 1** Changes in blood glucose and weight before and after treatment in EXO PBS groups. **Fig. 2** Identification of exosomes of hUCMSCs. **Fig. 3** Exosome therapy reduced the microvascular permeability of lower limb muscles in diabetic mice. **Fig. 4** Immunofluorescence staining of CD31 in muscle vessels and electron microscopy of capillaryultrastructure. **Fig. 5** Quantitative protemics by TMT and biochemistry analysis. **Fig. 6** Identification of CD105 and TGF-β1 proteins. **Fig. 7** Therapeutic mechanism of hUCMSCs after increased microcirculation permability in diabetic mice.

## Data Availability

Not applicable.
